# Interaction Mechanisms Among Soil Environmental Factors, Microbial Communities, and Nitrogen-Cycling Functional Genes in Cool-Climate Maize Fields

**DOI:** 10.3390/microorganisms14081705

**Published:** 2026-08-04

**Authors:** Qingqing Dai, Yuhang Wang, Mingji Jin, Shuo Wang, Mingji Han

**Affiliations:** 1 Agriculture College, Yanbian University, Yanji 133002, China; 2 College of Geography and Ocean Sciences, Yanbian University, Yanji 133002, China

**Keywords:** cool-climate maize fields, environmental factors, functional genes, microbial communities, nitrogen cycling

## Abstract

Cool-climate maize fields are characterized by low soil temperatures, strong seasonal hydrothermal fluctuations, and peat-influenced soil profiles, which may lead to patterns of nitrogen (N) cycling distinct from those in conventional agricultural soils. During maize growth, soils from three depths were characterized using physicochemical measurements, N-transformation and enzyme-activity assays, metagenomic sequencing, Mantel tests, variation partitioning analysis, and partial least squares path modeling (PLS-PM). Soil environmental factors varied significantly over time and with depth; soil organic matter (SOM) and total nitrogen (TN) increased with depth, while ammonium nitrogen (NH_4_^+^-N) predominated early and nitrate nitrogen (NO_3_^−^-N) predominated during the middle and late growth stages. The nitrogen fixation rate (NFR), nitrification rate (NitR), and denitrification rate (DNR) all peaked in August and showed a spatial pattern characterized by nitrogen fixation in the deepest layer and denitrification in the upper and middle layers. Bacterial communities varied less spatiotemporally than fungal communities. The genes *nifK*, *hao*, *nirS*/*nirK*, *NR*, *nrfC*, and *hzsA*/*hzsC* were identified as key nitrogen-cycling functional genes. Mantel tests and PLS-PM further characterized these relationships, with PLS-PM showing that soil physicochemical properties were positively associated with bacterial community composition (β = 0.87, *p* < 0.01), which, in turn, was negatively associated with N-cycling functional genes (β = −0.97, *p* < 0.001). Together, these pathways were associated with variation in N-cycling processes. Overall, this study advances an integrated understanding of N-cycling patterns and their potential controls in cool-climate maize fields and provides a scientific basis for optimizing N management strategies.

## 1. Introduction

Soil is fundamental to agricultural production and food security and is an irreplaceable natural resource with a very limited capacity for regeneration [1,2]. Among the various types of cropland, cool-climate croplands have attracted considerable attention because of their distinctive climatic conditions and ecological settings. Cool-climate croplands occur mainly at high latitudes or high elevations and experience pronounced climatic seasonality [3]. These regions typically have short, cool summers; long, cold winters; relatively low and unevenly distributed precipitation; and large diurnal temperature ranges, all of which pose substantial challenges to crop growth and farmland management [4]. In China, cool-climate croplands are concentrated primarily in Northeast China, Northwest China, and the Qinghai–Tibet Plateau [5]. Cool-climate maize fields in Northeast China, for example, are subject to seasonal freeze–thaw cycles and consequently experience pronounced fluctuations in soil temperature [6,7]. Maize (*Zea mays* L.) is one of the world’s most important food and feed crops and has relatively strong cold tolerance and high yield potential, making it an important crop in cool-climate croplands [8]. However, constrained by the cool climate, maize in Northeast China is generally sown in spring and completes its development primarily during the short period of suitable growing conditions in summer and early autumn; consequently, its growth period is considerably shorter than that of maize in southern China. Low soil temperatures not only restrict maize growth and development but may also alter nutrient supply patterns in cool-climate cropland ecosystems by affecting soil microbial metabolism, nutrient release processes, and microbially mediated elemental cycling.

Nitrogen (N) is a major soil nutrient and an essential component of amino acids, proteins, nucleic acids, and other biomolecules in crops, making it indispensable for crop growth and development [9]. In agroecosystems, N transformations are driven primarily by soil microorganisms. Different functional microbial groups collectively regulate the input, transformation, retention, and loss of soil N through biological nitrogen fixation, nitrification, denitrification, nitrate reduction, and anaerobic ammonium oxidation (anammox), thereby affecting the supply of plant-available N [10]. Soil N transformation processes are jointly influenced by environmental factors, including soil temperature (T), soil water content (SW), and pH, as well as microbial community structure [11]. Under the cold climatic conditions prevailing in cool-climate croplands, low soil temperatures influence soil chemical reaction rates, microbial activity, and crop N uptake and use efficiency, resulting in distinctive N-cycling dynamics [12]. Therefore, elucidating the relationships among environmental factors, microbial communities, and N-cycling functional genes in cool-climate maize fields is important for understanding N-cycling mechanisms in low-temperature agroecosystems and improving N use efficiency.

In recent years, researchers worldwide have investigated the effects of seasonal variation, soil depth, and agricultural management on soil microbial communities and N cycling. Regarding temporal variation, Gong et al. [13] found that freeze–thaw processes significantly altered the physicochemical properties, microbial community structure, and potential carbon and N metabolic functions of peat soils in the frozen-ground regions of Northeast China. Based on an in situ study of black-soil fields under continuous maize cropping in Northeast China, Deng et al. [14] further showed that seasonal freeze–thaw events, straw mulching, and N fertilizer management altered the response patterns and network stability of bacterial and fungal communities. Furthermore, using high-frequency monitoring over two consecutive years, Yang et al. [15] found that the abundances of N-cycling functional genes in cropland soils exhibited pronounced seasonal dynamics and were jointly influenced by soil total nitrogen (TN), precipitation, soil texture, and tillage practices. At the spatial scale, Bier et al. [16] showed that soil depth and fertility source were important factors shaping bacterial, archaeal, and fungal community composition across 0–60 cm cropland soil profiles and that the effects of different agricultural management practices on microbial communities varied with soil depth.

Overall, previous studies have shown that seasonal variation, soil depth, and agricultural management significantly influence soil microbial community structure and N-cycling processes in croplands. However, most studies have focused on a single environmental change or ecological process, and the integrated mechanisms regulating cropland soil N cycling under multiple interacting factors remain poorly understood [17]. This knowledge gap is particularly evident in cool-climate maize fields characterized by persistently low temperatures, pronounced hydrothermal fluctuations, and short growing seasons. It remains unclear how seasonal variation and vertical soil heterogeneity jointly shape microbial community structure and N-cycling functional gene composition and whether environmental factors indirectly regulate N transformation processes through their effects on microbial communities.

Against this background, this study investigated cool-climate maize fields by integrating measurements of soil physicochemical properties, N transformation rates, and key enzyme activities with analyses of microbial community composition and N-cycling functional genes to characterize the spatiotemporal dynamics of N-cycling processes and identify their potential drivers. Mantel tests, VPA, and PLS-PM were further used to examine the potential direct and indirect relationships among soil environmental factors, microbial communities, N-cycling functional genes, and N transformation processes.

This study aimed to address the following scientific questions: (1) How do soil environmental factors, microbial communities, and N-cycling functional genes vary across seasons and soil depths in cool-climate maize fields? (2) Which environmental factors are the key drivers of variation in microbial communities and N-cycling functional genes in these fields? (3) Do environmental factors potentially regulate N transformation processes indirectly through their effects on microbial communities and N-cycling functional genes?

## 2. Materials and Methods

### 2.1. Overview of the Study Area

**Figure 1:**
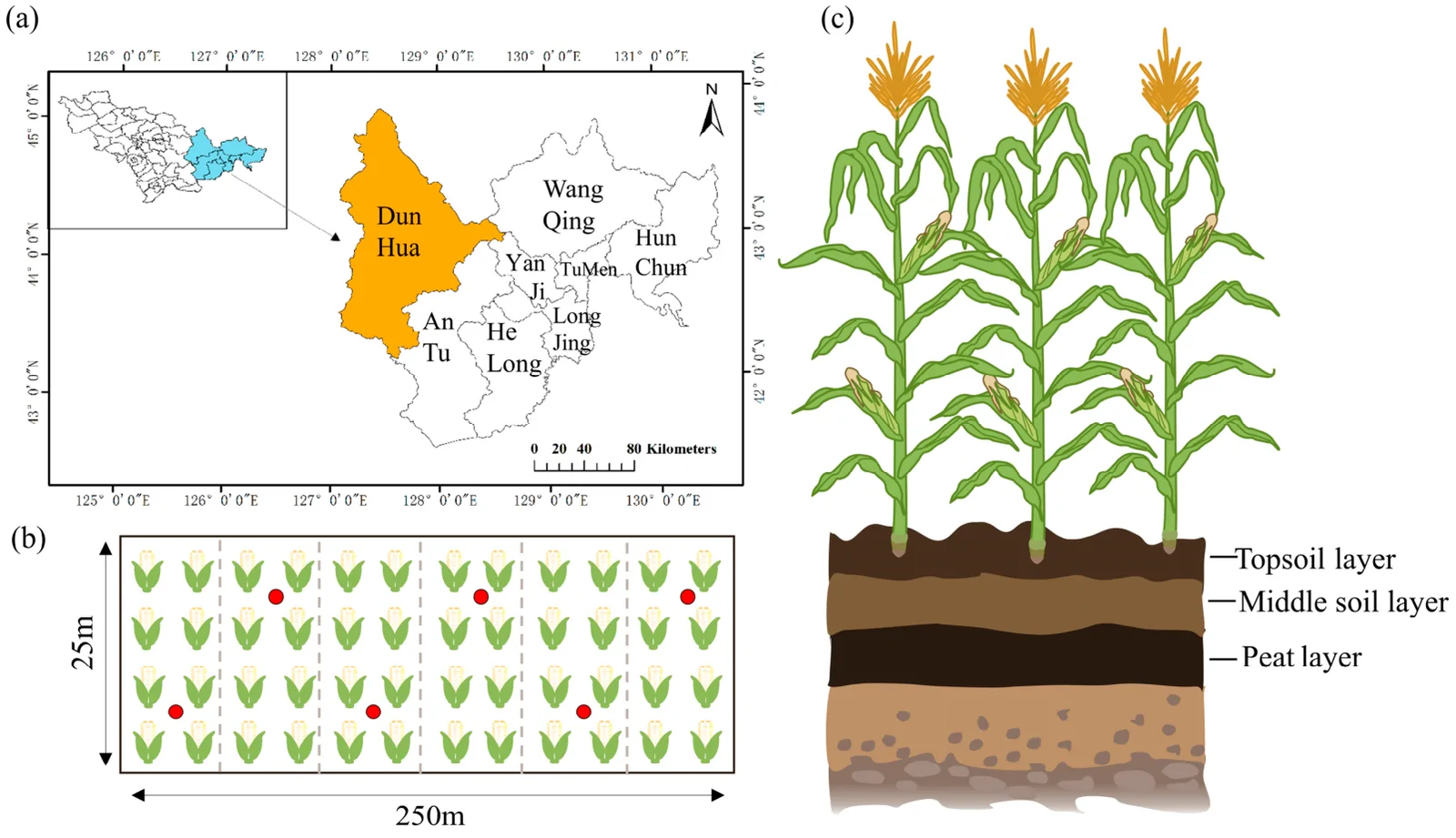
Location of the study area (**a**) and soil sampling scheme (**b**,**c**). The red circles indicate the soil sampling locations arranged according to the six-point sampling method.

As shown in Figure 1a, this study was conducted in a cool-climate maize field in Dunhua City, Jilin Province, China. The study site is located in Yujiashang Village, Daqiao Township, in the hilly area at the northern foot of the Harba Ridge (128°22′22″–128°22′23″ E, 43°18′46″–43°18′54″ N). The region has a typical temperate continental monsoon climate, characterized by long, cold winters and short, cool summers, with an annual mean temperature of 2.3–4.8 °C, and is one of the representative cool-climate agricultural regions in China [18]. The study area is underlain by a thick peat layer that thaws slowly in spring, resulting in persistently low soil temperatures that delay crop development and restrict crop growth [19]. A field under continuous maize cropping was selected for the experiment. Before maize planting each year, composted farmyard manure was applied uniformly at 15,000 kg ha^−1^, followed by topdressing with nitrogen, phosphorus, and potassium fertilizers at 375, 225, and 150 kg ha^−1^, respectively, during the maize growing season.

### 2.2. Sample Collection and Analysis

As shown in Figure 1b,c, soil samples were collected using a six-point sampling method [20] from the topsoil layer (0–20 cm), middle layer (20–40 cm), and peat layer (40–60 cm). Sampling was conducted once per month throughout the maize growing season from May to October. After collection, the soil samples were transported to the laboratory and divided into several portions. One portion was dried in the dark and protected from wind for the determination of soil physicochemical properties and N transformation rates. A second portion was stored frozen for the determination of soil enzyme activities. A third portion was combined in equal masses according to the corresponding analytical requirements and stored frozen for soil microbial community analysis. The remaining soil was used immediately to determine SW.

A total of 16 soil environmental factors were measured: pH, T, SW, soil organic matter (SOM), total nitrogen TN, nitrate nitrogen (NO_3_^−^-N), nitrite nitrogen (NO_2_^−^-N), ammonium nitrogen (NH_4_^+^-N), microbial biomass carbon (MBC), microbial biomass nitrogen (MBN), nitrogen fixation rate (NFR), nitrification rate (NitR), denitrification rate (DNR), nitrogenase activity (NA), Nitrite oxidase activity (NIO), and denitrifying enzyme activity (DE). The corresponding analytical methods are described in Table 1.

### 2.3. DNA Extraction and Metagenomic Sequencing

Total microbial DNA extraction, library preparation, and metagenomic sequencing were performed by Shanghai Personal Biotechnology Co., Ltd. (Shanghai, China). Total microbial DNA was extracted from the soil samples using the MagBeads FastDNA Kit for Soil (catalog no. 116564384; MP Biomedicals, Santa Ana, CA, USA) according to the manufacturer’s instructions and stored at −20 °C until further analysis. DNA concentration was determined using a Qubit™ 4 Fluorometer (Q33238), Qubit™ Assay Tubes (Q32856), and a Qubit™ 1X dsDNA HS Assay Kit (Q33231; Invitrogen, Life Technologies Corporation, Eugene, OR, USA), and DNA quality was assessed by 1% agarose gel electrophoresis. DNA samples that passed quality control were used to prepare sequencing libraries with the Illumina TruSeq Nano DNA LT Library Preparation Kit (Illumina, Inc., San Diego, CA, USA). The resulting libraries had an insert size of approximately 400 bp and were subjected to 150 bp paired-end (PE150) sequencing on an Illumina NovaSeq platform.

Raw sequencing reads were quality-filtered to obtain high-quality clean reads, which were used for subsequent assembly and gene prediction. The high-quality reads were assembled using MEGAHIT (v1.1.2), and contigs ≥ 300 bp in length were retained. Open reading frames (ORFs) were then predicted using Prodigal to obtain gene sequences and their corresponding protein sequences. The predicted gene sequences were clustered and dereplicated using MMseqs2 to construct a nonredundant gene catalog. The high-quality reads from each sample were mapped back to the nonredundant gene catalog, and gene abundances were calculated based on the mapping results. Gene abundances were calculated from the mapping results and normalized using the transcripts per million (TPM) method. N-cycling functional genes were annotated and screened using the Nitrogen Cycling Database (NCycDB), yielding information on their composition and abundance in each sample.

### 2.4. Statistical Analysis

IBM SPSS Statistics 27.0.1 (IBM Corp., Armonk, NY, USA) was used to perform two-way analysis of variance (ANOVA) on soil physicochemical properties, N transformation rates, and key enzyme activities and to assess the significance of the interaction between sampling month and soil layer. When significant effects were detected, pairwise comparisons were performed using Tukey’s honestly significant difference (HSD) test at *p* < 0.05.

In addition, Mantel tests based on Bray–Curtis dissimilarities were used to evaluate the associations among soil environmental factors, microbial communities, and N-cycling functional genes. Environmental factors showing significant associations (*p* < 0.05) were retained as explanatory variables for VPA. PCoA based on Bray–Curtis dissimilarities was also performed on the complete set of N-cycling functional genes, and the scores of the first two axes were used as response variables in VPA. VPA was performed using the vegan package in R.

PLS-PM analysis was performed using the plspm package in R. Soil physicochemical properties, N-cycling functional genes, N transformation rates, and key enzyme activities were represented through dimensionality reduction using principal component analysis (PCA), whereas bacterial community composition was represented through PCoA based on Bray–Curtis dissimilarities.

## 3. Results

### 3.1. Soil Environmental Factors

#### 3.1.1. Soil Physicochemical Properties

**Figure 2:**
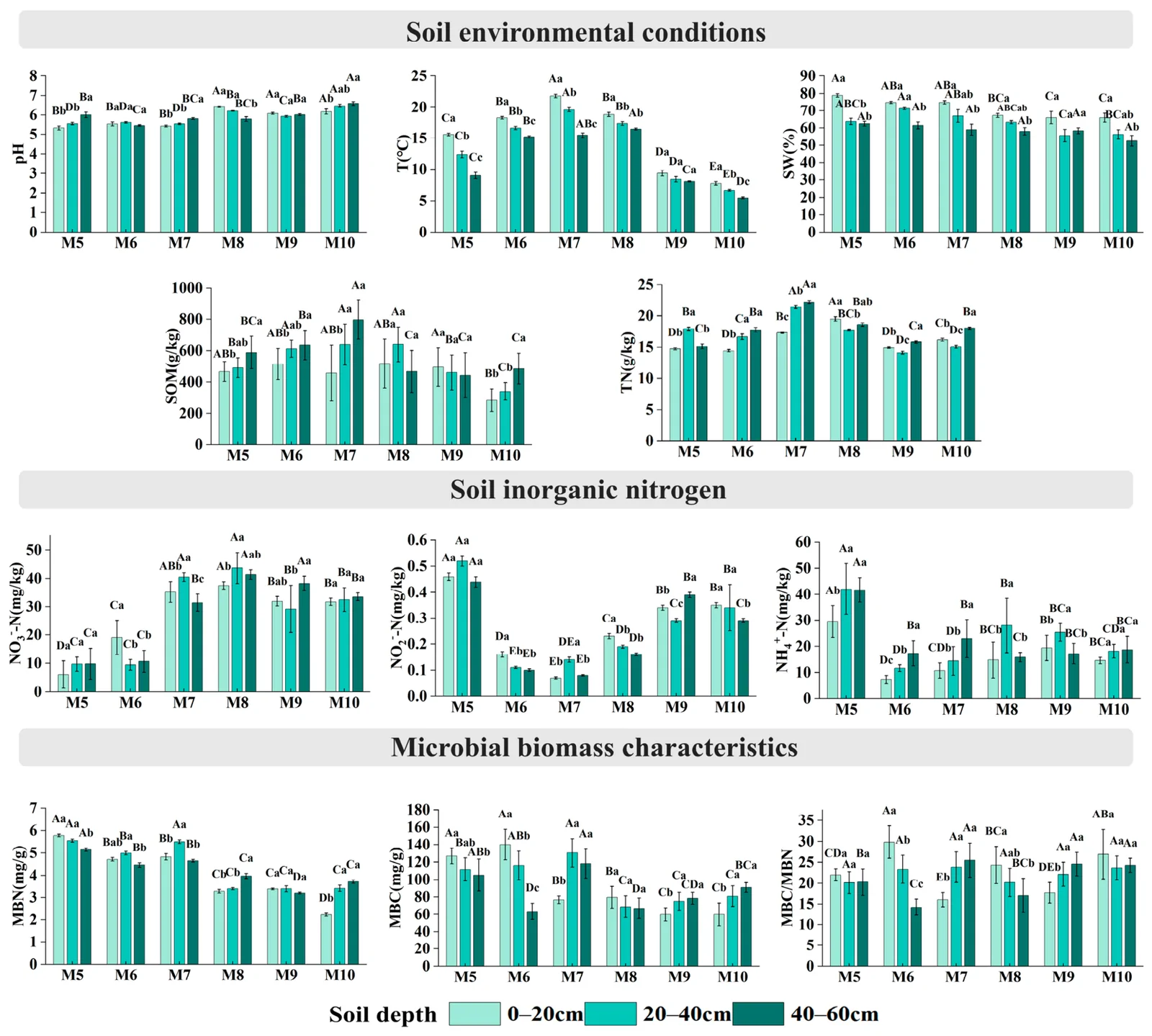
Soil physicochemical properties across different sampling months and soil layers. Uppercase letters indicate significant differences among sampling months within the same soil layer, and lowercase letters indicate significant differences among soil layers within the same sampling month (*p* < 0.05).

As shown in Figure 2, soil physicochemical properties exhibited significant temporal and spatial variation during the maize growing season. Temporally, soil pH remained mildly acidic throughout the study and increased slightly from August to October, although it remained below 6.5. Consistent with the local climatic pattern, T peaked in July, with the second-highest value observed in August, whereas SW was relatively high in May and June. SOM and TN contents both peaked in July.

Inorganic N forms, including NH_4_^+^-N, NO_3_^−^-N, and NO_2_^−^-N, differed significantly among months in association with fertilization and maize growth. NH_4_^+^-N and NO_2_^−^-N both peaked in May, when NH_4_^+^-N was the dominant form of soil inorganic N. From June onward, NO_3_^−^-N increased significantly and became the dominant inorganic N form, reaching its peak in August. MBC and MBN were relatively high from May to July, with both peaking in May. The MBC/MBN ratio was highest in October, suggesting that the microbial community may have experienced N limitation at this time. By contrast, this ratio was lowest in August, coinciding with the peak in NO_3_^−^-N, suggesting that C availability may have limited microbial activity during this period.

Regarding vertical variation, soil pH differed significantly among soil layers in all sampling months except June; T differed significantly among layers in all months except September; and SW differed significantly among layers in all months except August. Both T and SW decreased with increasing soil depth. SOM differed significantly among soil layers in all months except August and September, whereas TN differed significantly among layers in every sampling month. Both SOM and TN contents increased with soil depth.

In addition, NO_2_^−^-N and NO_3_^−^-N did not differ significantly among soil layers in May, nor did NH_4_^+^-N and NO_3_^−^-N in October; significant differences among soil layers were detected for the other inorganic N measurements. Inorganic N concentrations were relatively higher in the middle and peat layers than in the topsoil layer, potentially reflecting downward leaching and accumulation.

MBC and MBN contents were also relatively higher in the middle layer than in the other soil layers. MBC differed significantly among soil layers in all months except August, whereas MBN differed significantly among layers in all months except September. The MBC/MBN ratio was higher in the topsoil layer, possibly because of greater inputs of readily decomposable C substrates. By contrast, persistently anaerobic conditions in the peat layer may have favored microorganisms with relatively low biomass C/N ratios, potentially contributing to the lower MBC/MBN ratio in this layer.

#### 3.1.2. Soil Nitrogen Transformation Rates and Key Enzyme Activities

**Figure 3:**
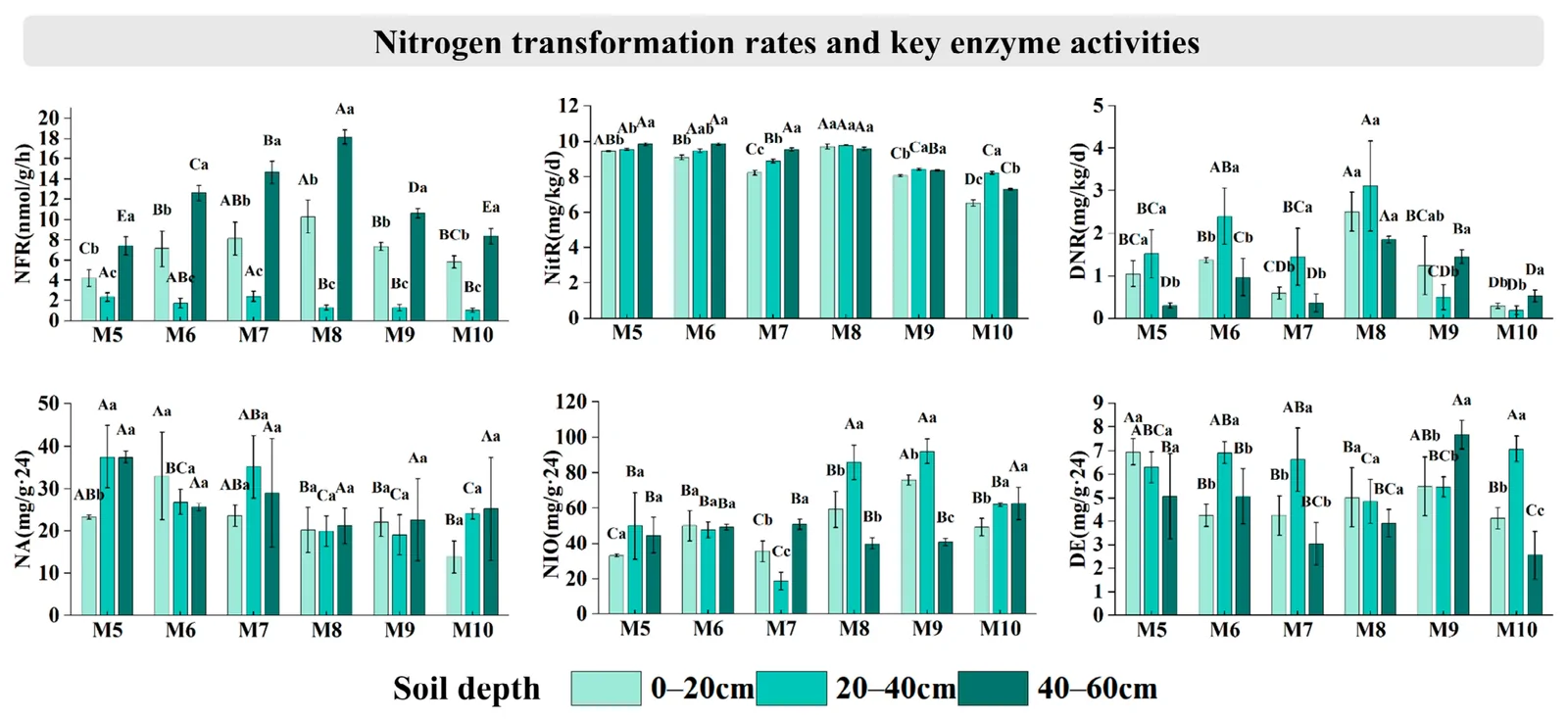
Nitrogen transformation rates and key enzyme activities across different sampling months and soil layers. Uppercase letters indicate significant differences among sampling months within the same soil layer, and lowercase letters indicate significant differences among soil layers within the same sampling month (*p* < 0.05).

As shown in Figure 3, soil N transformation rates and key enzyme activities exhibited significant temporal and spatial variation. Temporally, NFR, NitR, and DNR all peaked in August. NA showed an overall decreasing trend and was highest in May, whereas NIO and DE peaked in September.

Regarding vertical variation, NFR was highest in the peat layer, followed by the topsoil layer, whereas NA did not differ significantly among soil layers in most months. DNR and DE were generally higher in the topsoil and middle layers than in the peat layer, suggesting that denitrification was more pronounced in the upper and middle layers. In addition, NIO was highest in the middle layer, suggesting greater potential for nitrite oxidation in this layer. Overall, the soil profile exhibited a complementary spatial pattern characterized by N fixation in the deepest layer and denitrification in the upper and middle layers.

### 3.2. Microbial Community Composition

Microbial communities were analyzed by sampling month and soil layer. Samples collected in May, June, July, September, and October were designated M5, M6, M7, M9, and M10, respectively. In addition, August, when microbial activity was relatively high, was selected for the analysis of microbial communities across different soil layers. Samples collected from the topsoil, middle, and peat layers in August were designated S8, D8, and P8, respectively.

#### 3.2.1. Bacterial Community Composition

**Figure 4:**
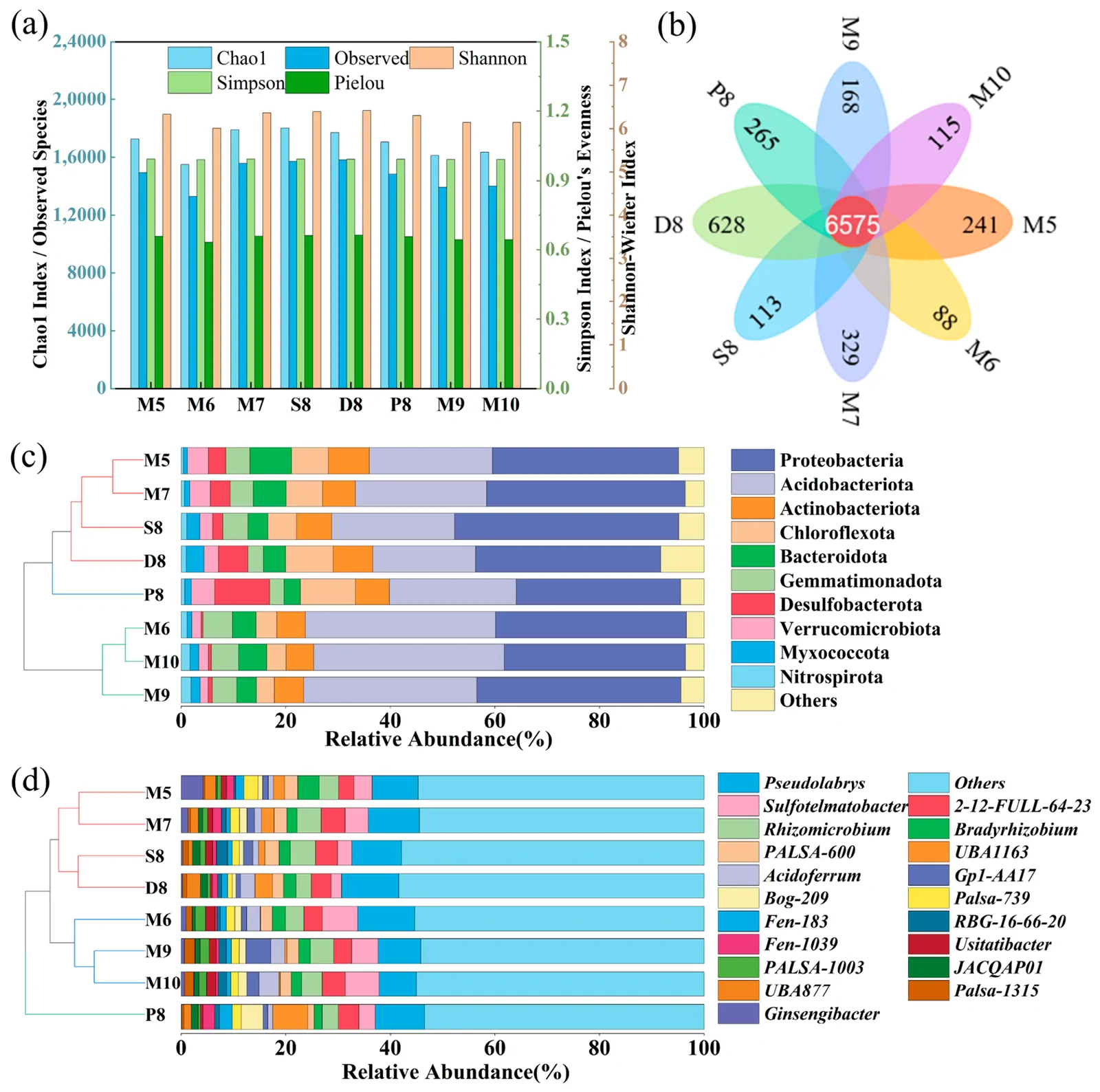
Bacterial community composition. (**a**) Bacterial community diversity; (**b**) petal diagram of bacterial species; (**c**) bacterial community structure at the phylum level; (**d**) bacterial community structure at the genus level.

As shown in Figure 4a, the maximum values of Chao1 richness, observed species richness, the Shannon index, the Simpson index, and Pielou’s evenness index for bacterial communities occurred in S8, D8, D8, M7, and D8, respectively, whereas all five indices reached their minimum values in M6. Overall, bacterial alpha diversity approximately followed the order D8 > S8 > M7 > M5 > P8 > M10 > M9 > M6. August corresponded to a period of vigorous crop growth in the study area, when relatively high soil temperatures and microbial activity may have contributed to the generally high bacterial diversity. Across soil layers, the intermediate redox conditions in the middle layer may support the coexistence of aerobic and anaerobic microorganisms, thereby helping maintain relatively high bacterial diversity.

As shown in Figure 4b, all samples shared 6575 bacterial species, accounting for 50.23%, 56.39%, 47.57%, 50.37%, 45.73%, 51.35%, 55.58%, and 54.12% of the total species detected in M5, M6, M7, S8, D8, P8, M9, and M10, respectively. In contrast, species unique to these samples accounted for only 1.84%, 0.75%, 2.38%, 0.87%, 4.37%, 2.07%, 1.42%, and 0.94% of their respective total species. These results indicate a high overall similarity in bacterial community composition among samples, with relatively limited variation across sampling months and soil layers. In addition, D8 contained more unique species than the other samples, which may be associated with the intermediate redox conditions in the middle layer that potentially support the coexistence of aerobic and anaerobic microorganisms.

As shown in Figure 4c, at the phylum level, Proteobacteria, Acidobacteriota, Actinobacteriota, Chloroflexota, Bacteroidota, and Gemmatimonadota were the dominant bacterial phyla. Together, these six phyla accounted for more than 78% of the bacterial community in every sample. Among them, the relative abundances of Chloroflexota and Bacteroidota showed greater variation across sampling months, whereas those of Gemmatimonadota and Chloroflexota varied more markedly among soil layers.

As shown in Figure 4d, at the genus level, *Pseudolabrys*, *Sulfotelmatobacter*, 2-12-FULL-64-23, *Rhizomicrobium*, *Bradyrhizobium*, and PALSA-600 were the dominant bacterial genera. Among them, the relative abundances of *Sulfotelmatobacter* and *Bradyrhizobium* showed greater variation across sampling months. *Sulfotelmatobacter* was relatively more abundant in June and October than in the other months, whereas *Bradyrhizobium* was relatively more abundant in May. PALSA-600 and *Rhizomicrobium* showed greater variation among soil layers.

As shown in Figure 4c,d, at both the phylum and genus levels, M5, M7, S8, and D8 formed one major cluster, P8 formed a separate cluster, and M6, M9, and M10 formed another major cluster. Thus, bacterial community structures were relatively similar in May, July, and August, as were those in June, September, and October, whereas the bacterial community in the peat layer formed a separate cluster, possibly because of its distinct anaerobic environment.

#### 3.2.2. Fungal Community Composition

**Figure 5:**
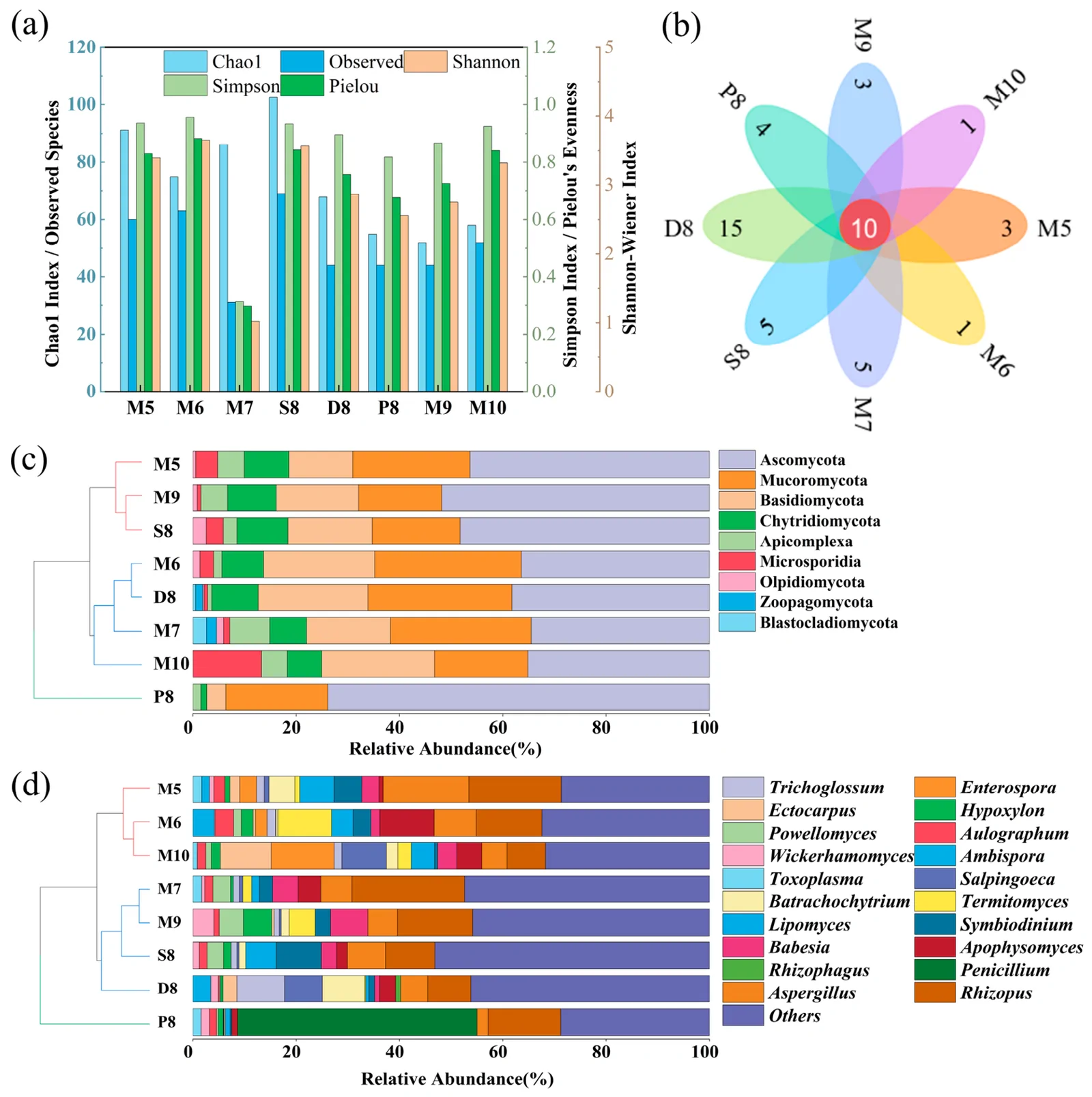
Fungal community composition. (**a**) Fungal community diversity; (**b**) petal diagram of fungal species; (**c**) fungal community structure at the phylum level; (**d**) fungal community structure at the genus level.

As shown in Figure 5a, the maximum values of Chao1 richness, observed species richness, the Shannon index, the Simpson index, and Pielou’s evenness index for fungal communities occurred in S8, S8, M6, M6, and M6, respectively, whereas the minimum values were predominantly observed in M7. Overall, fungal alpha diversity approximately followed the order M6 > S8 > M5 > M10 > D8 > M9 > P8 > M7, which differed markedly from the ranking of bacterial alpha diversity. Considering bacterial and fungal community diversity together, both were generally high in S8 in August, possibly reflecting the climatic conditions and crop growth in the study area.

As shown in Figure 5b, all samples shared 10 fungal species, accounting for 13.89%, 15.87%, 15.15%, 13.89%, 10.99%, 17.24%, 16.67%, and 18.52% of the total fungal species detected in M5, M6, M7, S8, D8, P8, M9, and M10, respectively. These relatively low proportions of shared species indicate limited similarity in fungal species composition among samples and substantial variation in fungal community composition across sampling months and soil layers.

As shown in Figure 5c, at the phylum level, Ascomycota, Mucoromycota, Basidiomycota, and Chytridiomycota were the dominant fungal phyla, with a combined relative abundance exceeding 80% in every sample. Among them, the relative abundances of Basidiomycota and Mucoromycota showed greater variation across sampling months, whereas those of Basidiomycota and Chytridiomycota varied more markedly among soil layers.

As shown in Figure 5d, *Rhizopus* was the most abundant fungal genus in M5, M6, M7, S8, and M9, with relative abundances of 17.88%, 12.72%, 21.82%, 9.54%, and 14.56%, respectively. In D8, P8, and M10, the most abundant fungal genera were *Trichoglossum*, *Penicillium*, and *Enterospora*, with relative abundances of 9.23%, 46.38%, and 12.15%, respectively. Among these genera, *Enterospora* showed greater variation across sampling months, whereas *Penicillium* was detected only in P8, indicating marked variation among soil layers.

As shown in Figure 5c,d, at the phylum level, M5, M9, and S8 formed one major cluster, P8 formed a separate cluster, and M6, M7, D8, and M10 formed another major cluster. At the genus level, M5, M6, and M10 formed one major cluster, P8 formed a separate cluster, and M7, M9, S8, and D8 formed another major cluster. Taken together, the phylum- and genus-level results showed that the fungal community in the peat layer formed a separate cluster, possibly reflecting the distinct anaerobic conditions in this layer. This pattern was consistent with the bacterial community clustering results.

### 3.3. Nitrogen-Cycling Functional Genes

**Figure 6:**
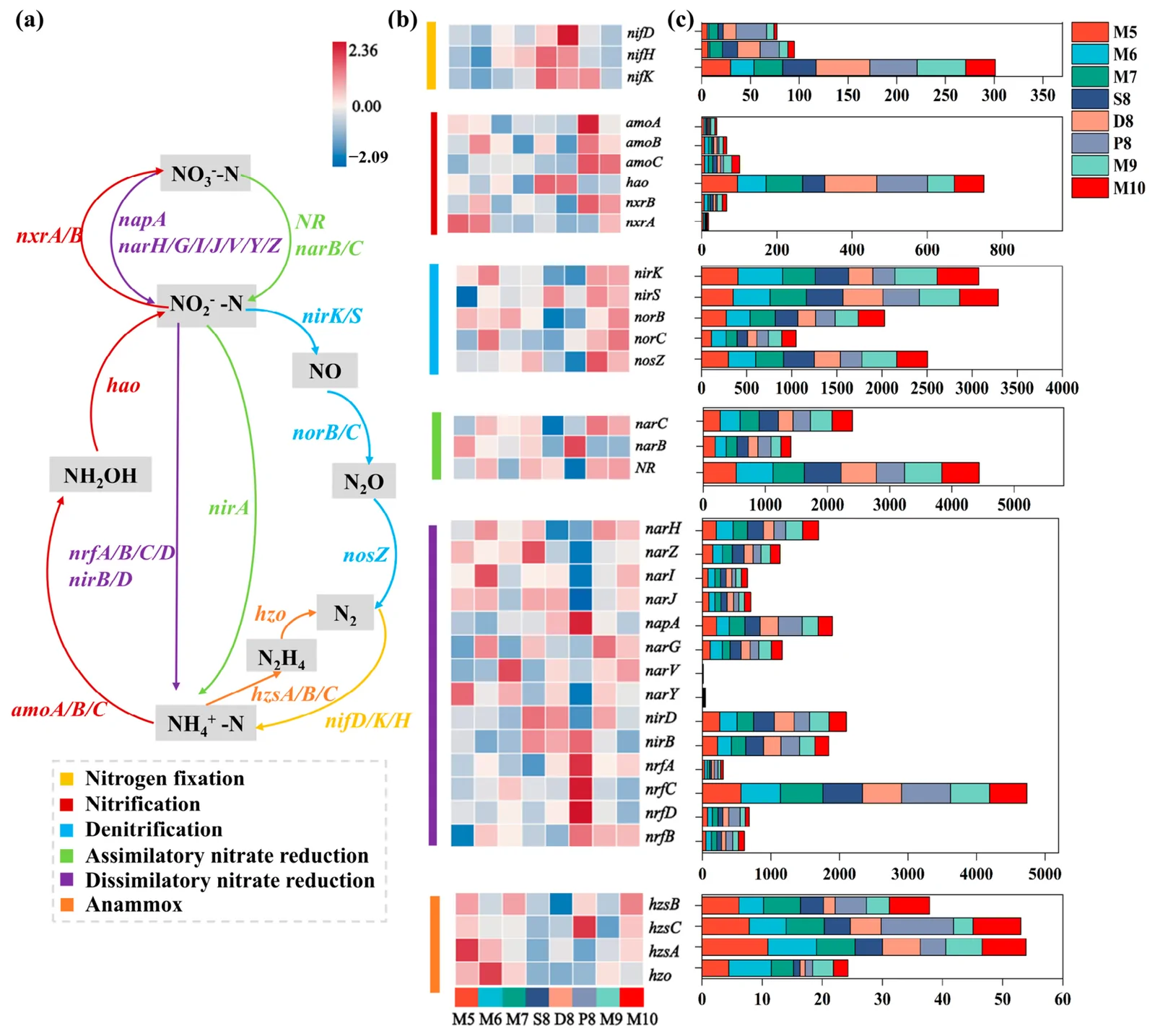
Nitrogen-cycling pathways and functional genes. (**a**) Nitrogen-cycling pathways; (**b**) heatmap of functional genes; (**c**) TPM values of functional genes.

As shown in Figure 6a, the soil N-cycling pathways examined in this study were classified as nitrogen fixation (NF), nitrification, denitrification, assimilatory nitrate reduction (ANR), dissimilatory nitrate reduction to ammonium (DNRA), and anammox. The detected genes included *nifD*, *nifH*, and *nifK* for *NF*; *amoA*, *amoB*, *amoC*, *hao*, *nxrA*, and *nxrB* for nitrification; *nirS*, *nirK*, *norB*, *norC*, and *nosZ* for denitrification; *narB*, *narC*, and *NR* for ANR; *narH*, *narG*, *narI*, *narJ*, *narV*, *narY*, *narZ*, *napA*, *nirB*, *nirD*, *nrfA*, *nrfB*, *nrfC*, and *nrfD* for DNRA; and *hzsA*, *hzsB*, *hzsC*, and *hzo* for anammox. As shown in Figure 6c, *nifK*, *hao*, *nirS*/*nirK*, *NR*, *nrfC*, and *hzsA*/*hzsC* were identified as key functional genes for NF, nitrification, denitrification, ANR, DNRA, and anammox, respectively. Their TPM-normalized relative abundances were higher than those of other functional genes involved in the corresponding pathways.

As shown in Figure 6b, genes involved in NF showed relatively high abundances in D8 and P8. Among the genes involved in nitrification, *amoA*, *amoB*, *amoC*, and *nxrB* were relatively abundant in M9 and M10; *hao* was relatively abundant in D8 and P8; and *nxrA* was relatively abundant in M5 and M6. All detected genes involved in denitrification showed relatively high abundances in M9 and M10. Notably, *nirS* showed a relatively low abundance in M5.

Among the genes involved in ANR, *narC* and *NR* showed relatively high abundances in M9 and M10, whereas *narB* was relatively abundant in P8. Among the genes involved in DNRA, *nrfA*, *nrfC*, *nrfD*, and *napA* showed relatively high abundances in P8. By contrast, *narI*, *narJ*, *narY*, and *narZ* showed relatively low abundances in P8. In addition, *narZ* was relatively abundant in S8, whereas *narV* was relatively abundant in M7. Among the genes involved in anammox, *hzsB* showed a relatively low abundance in D8, whereas *hzsC*, *hzsA*, and *hzo* showed relatively high abundances in P8, M5, and M6, respectively.

### 3.4. Correlation Analysis Among Soil Environmental Factors, Microbial Communities, and Nitrogen-Cycling Functional Genes

**Figure 7:**
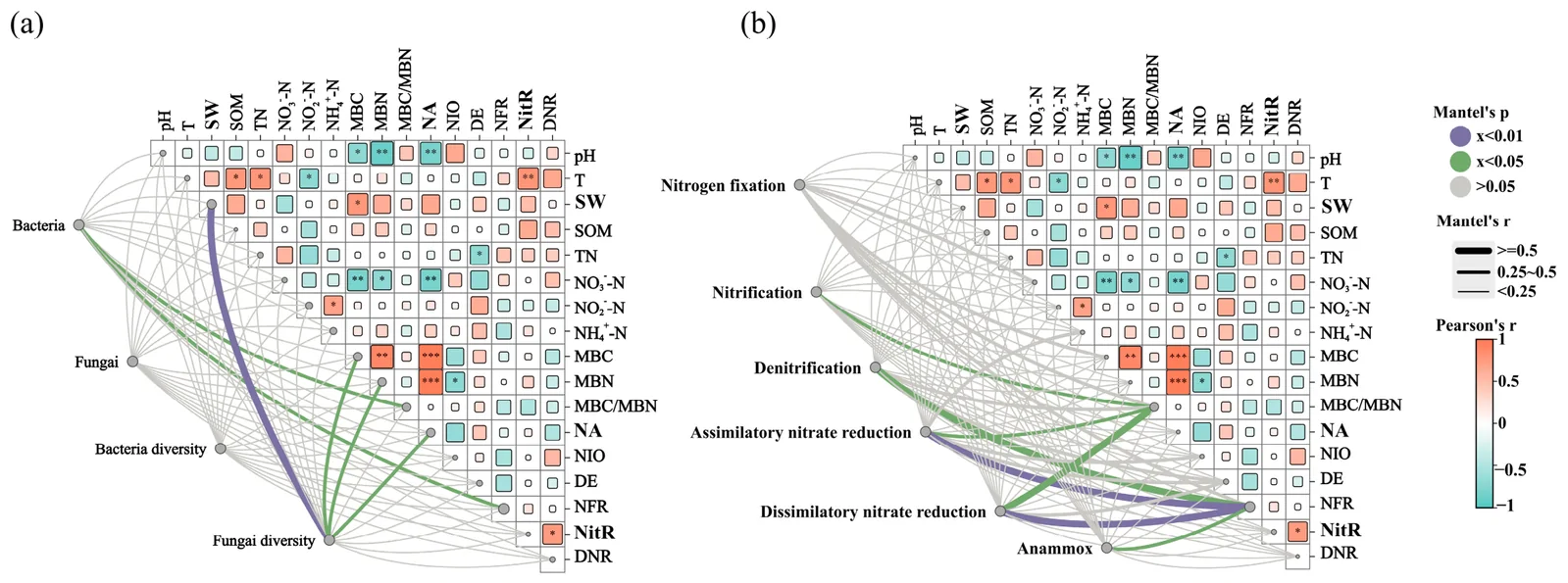
Mantel-test associations between soil environmental factors and (**a**) microbial communities and (**b**) N-cycling functional genes. *, **, and *** indicate *p* < 0.05, *p* < 0.01, and *p* < 0.001, respectively. The colors and sizes of the squares represent the direction and absolute magnitude of Pearson’s r, respectively.

To further investigate the associations among soil environmental factors, microbial communities, and N-cycling functional genes, Mantel tests were performed. As shown in Figure 7a, bacterial community composition showed significant positive correlations with the MBC/MBN ratio and NFR (r = 0.47 and 0.42, respectively; *p* < 0.05). Fungal community diversity showed significant positive correlations with MBC, MBN, and NA (r = 0.47, 0.33, and 0.37, respectively; *p* < 0.05) and a significant positive correlation with SW (r = 0.59; *p* < 0.01).

As shown in Figure 7b, the MBC/MBN ratio showed significant positive correlations with genes involved in nitrification, ANR, and DNRA (r = 0.46, 0.38, and 0.63, respectively; *p* < 0.05). NFR showed significant positive correlations with genes involved in denitrification and anammox (r = 0.50 and 0.38, respectively; *p* < 0.05) and significant positive correlations with genes involved in ANR and DNRA (r = 0.58 and 0.78, respectively; *p* < 0.01). Overall, MBC, MBN, SW, NA, the MBC/MBN ratio, and NFR were the principal soil environmental factors associated with microbial communities and N-cycling functional genes in cool-climate maize fields.

**Figure 8:**
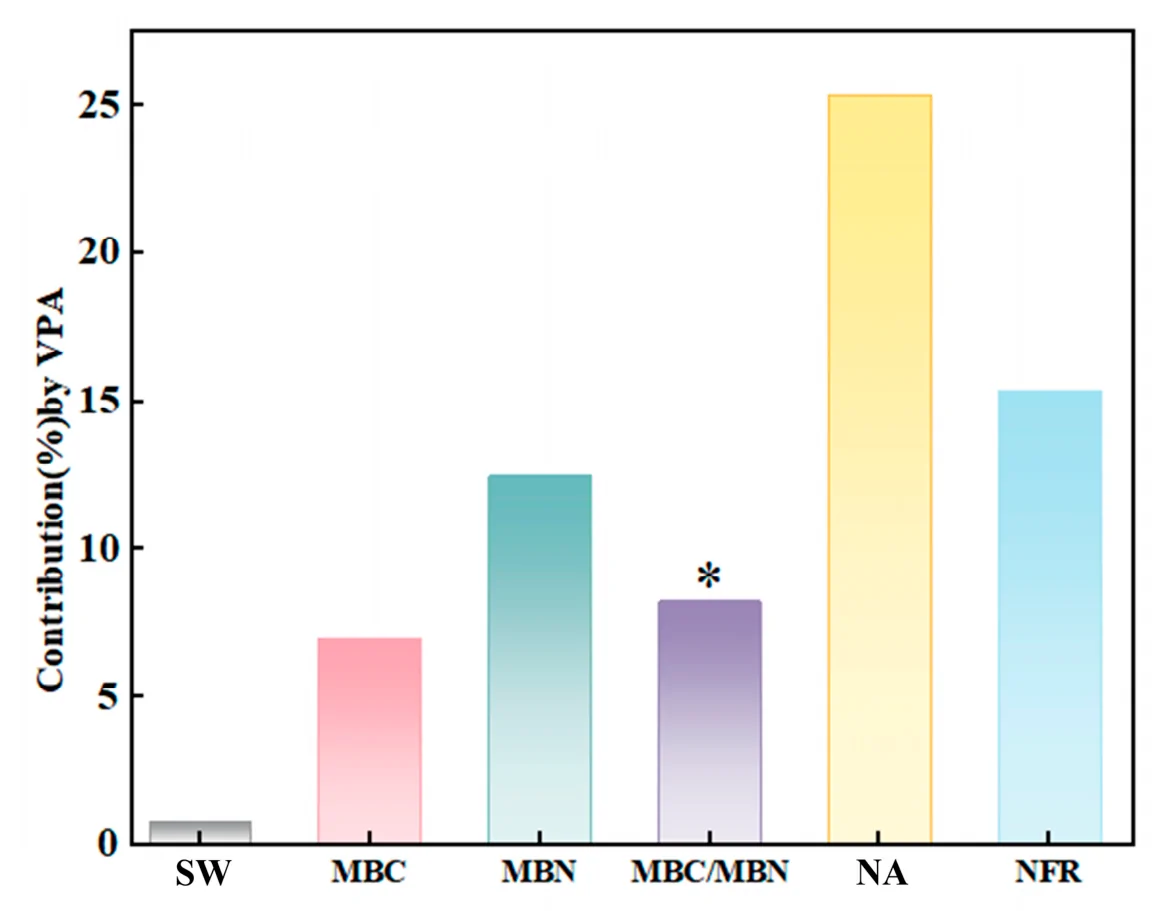
Variation partitioning analysis of the contributions of the principal soil environmental factors to variation in N-cycling functional gene composition. An asterisk indicates statistical significance at *p* < 0.05.

Based on the principal soil environmental factors identified above, including MBC, MBN, SW, NA, the MBC/MBN ratio, and NFR, VPA was further performed to quantify the proportion of variation in N-cycling functional gene composition explained by each variable. As shown in Figure 8, these variables collectively explained 99.2% of the total variation in N-cycling functional gene composition. Among them, NA (25.34%), NFR (15.39%), and MBN (12.48%) had relatively high unique explanatory fractions. Although the MBC/MBN ratio independently explained only 8.42% of the variation, its unique explanatory contribution was statistically significant, indicating that it independently accounted for a significant proportion of the variation in N-cycling functional gene composition.

**Figure 9:**
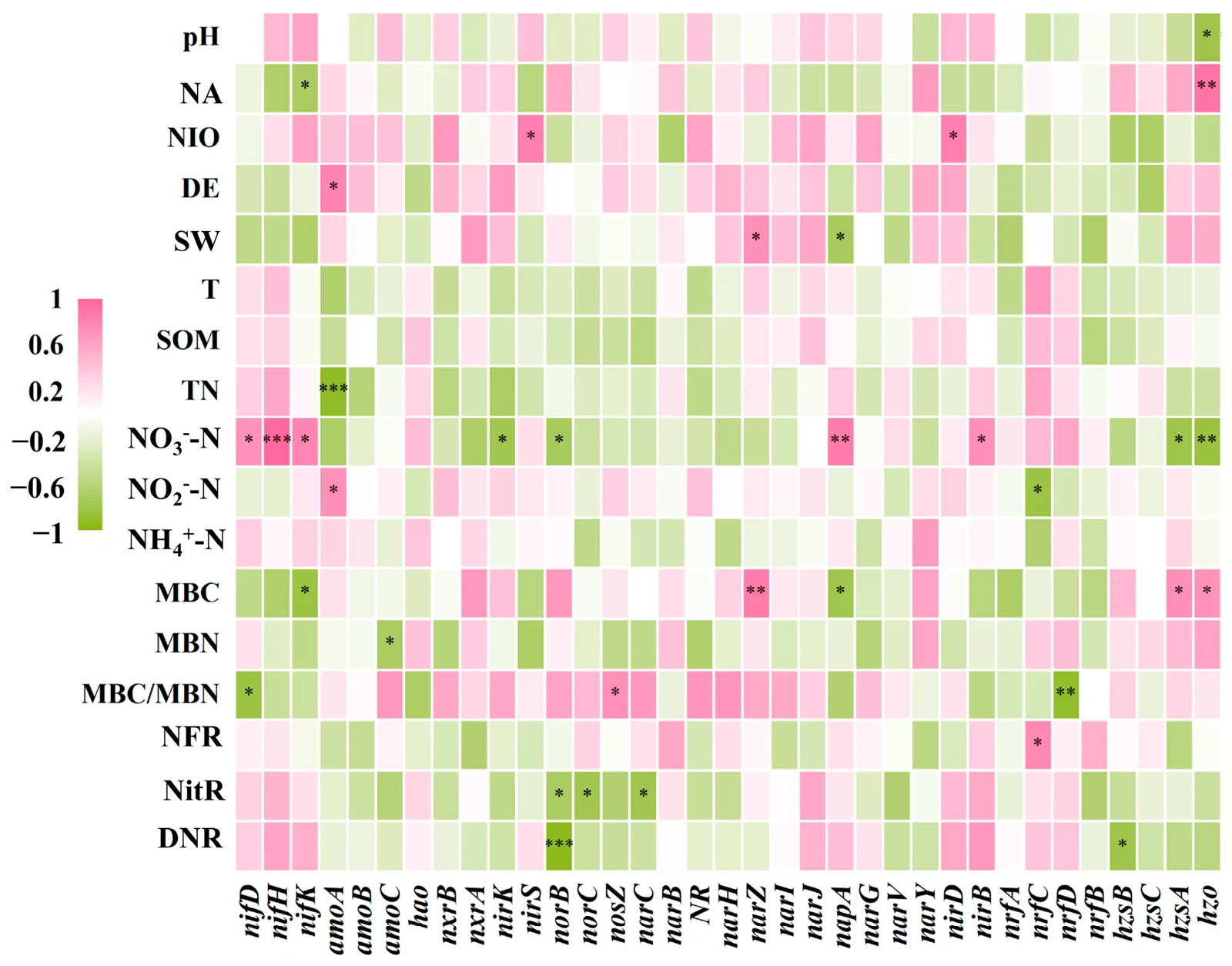
Correlations between soil environmental factors and nitrogen-cycling functional genes. *, **, and *** indicate *p* < 0.05, *p* < 0.01, and *p* < 0.001, respectively.

Further correlation analyses were performed to examine the associations between soil environmental factors and N-cycling functional genes (Figure 9). Among the genes involved in NF, *nifD*, *nifH*, and *nifK* showed significant positive correlations with NO_3_^−^-N. *nifD* showed a significant negative correlation with the MBC/MBN ratio, whereas *nifK* showed significant negative correlations with NA and MBC. Among the genes involved in nitrification, *amoA* showed a significant negative correlation with TN but significant positive correlations with NO_2_^−^-N and DE, while *amoC* showed a significant negative correlation with MBN.

Among the genes involved in denitrification, *nosZ* showed a significant positive correlation with the MBC/MBN ratio, and *nirS* showed a significant positive correlation with NIO. Both *norB* and *norC* showed significant negative correlations with NitR; *norB* and *nirK* showed significant negative correlations with NO_3_^−^-N; and *norB* showed a significant negative correlation with DNR.

Among the genes involved in ANR, only *narC* showed a significant negative correlation with NitR. Among the genes involved in DNRA, *narZ* and *napA* showed significant positive correlations with MBC and NO_3_^−^-N, respectively. In addition, *narZ*, *nirD*, *nirB*, and *nrfC* showed significant positive correlations with SW, NIO, NO_3_^−^-N, and NFR, respectively. *nrfD* showed a significant negative correlation with the MBC/MBN ratio, while *napA* showed significant negative correlations with SW and MBC, and *nrfC* showed a significant negative correlation with NO_2_^−^-N.

Among the genes involved in anammox, *hzsA* and *hzo* showed significant positive correlations with MBC but significant negative correlations with NO_3_^−^-N. In addition, *hzo* showed a significant positive correlation with NA and a significant negative correlation with pH, whereas *hzsB* showed a significant negative correlation with DNR.

Overall, NO_3_^−^-N showed the strongest associations with N-cycling functional genes. MBC, the MBC/MBN ratio, NitR, and DNR were also closely associated with these genes. Among the functional genes, *nifK*, *amoA*, *norB*, *napA*, and *hzo* showed relatively strong associations with soil environmental factors.

**Figure 10:**
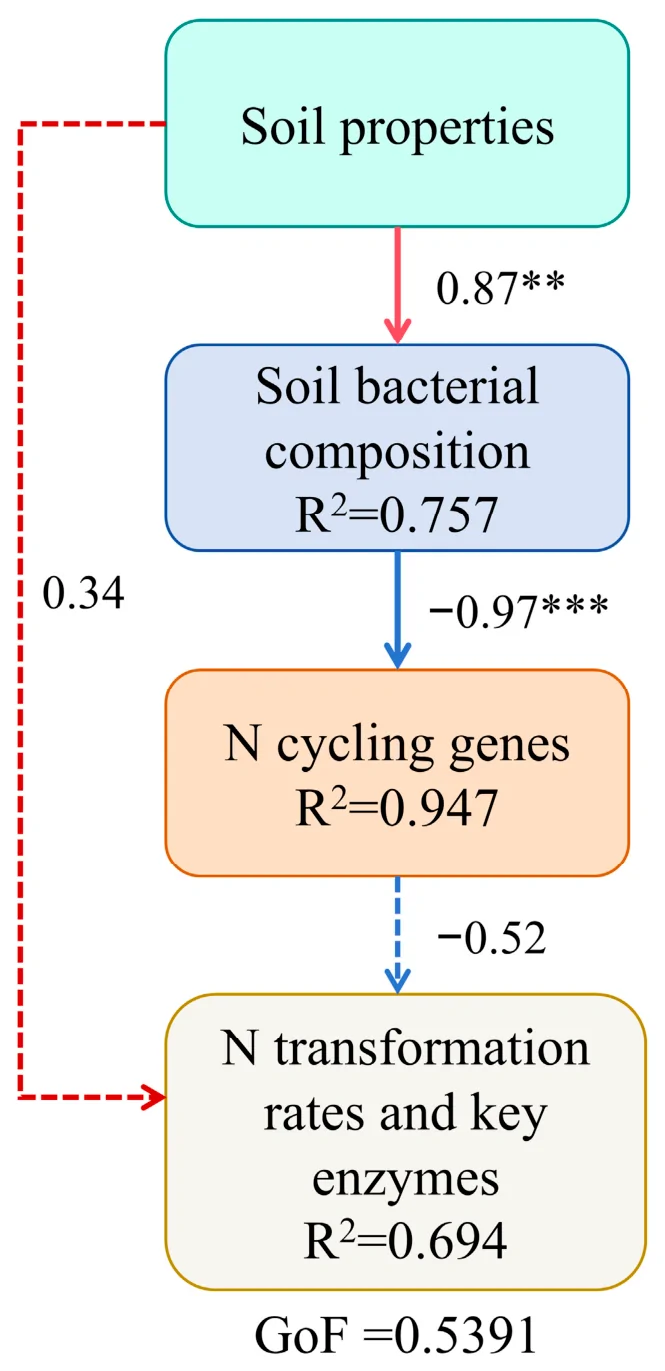
PLS-PM analysis of soil physicochemical properties, bacterial communities, nitrogen-cycling functional genes, nitrogen transformation rates, and key enzyme activities. Red and blue arrows indicate positive and negative relationships, respectively. Solid and dashed arrows indicate significant and non-significant paths, respectively. Numbers beside arrows indicate standardized path coefficients (β), and R^2^ values represent the explained variance. Asterisks indicate significance levels: **, and *** indicate *p* < 0.01, and *p* < 0.001, respectively.

In addition, PLS-PM was used to quantify the strength of the relationships among soil physicochemical properties, N transformation rates and key enzyme activities, bacterial community composition, and N-cycling functional genes in cool-climate maize fields. As shown in Figure 10, the model had a goodness of fit (GoF) of 0.5391, indicating a good overall fit. The model explained 75.7% of the variation in bacterial community composition, 94.7% of the variation in N-cycling functional genes, and 69.4% of the variation in N transformation rates and key enzyme activities.

Path analysis showed that soil physicochemical properties had a significant positive effect on bacterial community composition (β = 0.87, *p* < 0.01), whereas bacterial community composition had a significant negative effect on N-cycling functional genes (β = −0.97, *p* < 0.001). In addition, soil physicochemical properties had a positive direct effect on N transformation rates and key enzyme activities (β = 0.34), whereas N-cycling functional genes had a negative effect on these variables (β = −0.52). Overall, although bacterial community composition had a negative effect on N-cycling functional genes and N-cycling functional genes had a negative effect on N transformation rates and key enzyme activities, the indirect-effect analysis showed that soil physicochemical properties had a positive indirect effect on N transformation rates and key enzyme activities (indirect effect = 0.44). This indirect effect was stronger than the corresponding direct effect. These results indicate that N transformation rates and key enzyme activities were influenced primarily through the indirect pathway.

## 4. Discussion

### 4.1. Spatiotemporal Evolution of Soil Environmental Factors

The cool-climate maize fields investigated in this study were influenced by the underlying peat layer, and SOM content increased progressively with soil depth. Because soil organic N is primarily derived from SOM, TN content exhibited a similar increase with depth. The parallel increases in SOM and TN reflected the characteristic vertical distribution of peat-influenced soils. Soil inorganic N forms exhibited pronounced seasonal variation: NH_4_^+^-N predominated during the early stage of maize growth, whereas NO_3_^−^-N became the dominant form during the middle and late growth stages. This shift suggests that rising air temperatures in the study area may have enhanced nitrification, resulting in the progressive oxidation of ammonium to nitrate [24]. NO_2_^−^-N is an unstable intermediate of nitrification. Its consistently low content in the cool-climate maize fields examined here was consistent with its transient nature.

Soil MBC and MBN were both relatively high during the early stage of maize growth but gradually declined during the middle and late growth stages, potentially reflecting the continued depletion of soil C and N resources as maize growth progressed [25]. The soil MBC/MBN ratio exhibited pronounced seasonal variation, reaching its minimum in August and its maximum in October. This pattern suggests that microbial communities in the cool-climate maize fields may have been primarily limited by C availability in August and gradually shifted toward N limitation by October, possibly because of autumn inputs of litter with a high C/N ratio [26]. The relatively high MBC/MBN ratio in autumn further suggests that inputs of fresh external organic matter may strongly influence microbial biomass stoichiometry in cool-climate maize fields. This interpretation is consistent with the view proposed by Berg et al. [27] that litter quality shapes microbial metabolic strategies.

The timing of peak N transformation rates differed from that of some key enzyme activities, suggesting that these variables may respond differently to immediate environmental conditions and microbial functional adjustments in cool-climate maize fields. N transformation rates may respond rapidly to environmental factors such as soil temperature, moisture, and substrate availability, and they peaked in August. By contrast, NIO and DE may undergo functional adjustments in response to changes in intermediate substrate availability, with both peaking in September. This one-month delay suggests a lagged response in the regulation of these enzyme activities [28]. Collectively, these results indicate that N-cycling processes in cool-climate maize fields may be jointly shaped by short-term environmental variation and microbial functional adjustments.

### 4.2. Spatiotemporal Response Characteristics of Microbial Communities

Finn et al. [29] found in a long-term field experiment that prokaryotic community composition remained relatively stable during the crop growing season, whereas fungal communities exhibited more pronounced seasonal variation, indicating that fungi were more sensitive to environmental changes during the growing season. Consistent with their findings, bacterial and fungal community diversity and composition in the present study also responded differently to temporal and vertical gradients. For bacterial communities, unique species accounted for less than 4.4% of the species in each sample, whereas species shared among all samples accounted for 45–56% of the total species in each sample. These results indicate that bacterial communities exhibited high similarity and limited spatiotemporal variation. By contrast, fungal community diversity peaked in June and in the August topsoil sample. Fungal communities also exhibited greater fluctuations in diversity, higher compositional dissimilarity, and greater sensitivity to environmental variation.

Bacteria possess strong dispersal capacity, considerable physiological plasticity, and diverse metabolic strategies, enabling them to adapt to changes in temperature, moisture, and nutrient availability by adjusting their resource-use strategies [30,31]. Therefore, despite pronounced seasonal fluctuations in temperature and moisture and marked environmental differences among soil layers in the cool-climate maize fields, bacterial communities maintained a high degree of similarity. This pattern may reflect their strong capacity to adapt to environmental variation.

Compared with bacteria, many soil fungi grow as mycelial networks or occur as relatively large spores, which may limit their dispersal through the soil matrix and reduce efficient colonization over large spatial scales [32]. Their relatively long generation times and slow population turnover may also constrain rapid recovery and the re-establishment of stable communities following environmental disturbance [33]. In addition, fungal growth and metabolism are highly dependent on substrate availability, making fungi more sensitive to changes in C sources and nutrients [34]. Their environmental adaptability and metabolic flexibility may therefore be relatively limited, resulting in a lower capacity to buffer environmental fluctuations than that of bacteria [35]. Cool-climate maize fields are characterized by relatively low temperatures and variable moisture conditions. These pronounced environmental fluctuations may restrict the effective dispersal and stable establishment of fungi, thereby contributing to greater fluctuations in fungal diversity and more pronounced differences in species composition.

### 4.3. Spatiotemporal Differentiation Patterns of Nitrogen-Cycling Functional Genes

Previous research has shown that N-cycling functional genes in agricultural soils exhibit pronounced seasonal dynamics and that their variation is closely associated with temperature, moisture, N availability, and soil resource availability [15]. Low temperatures can constrain microbial metabolism and alter soil redox conditions. Accordingly, N-cycling functional gene profiles in the cool-climate maize fields examined here also exhibited distinct seasonal and soil-depth-related patterns.

The N-fixation genes *nifD*, *nifH*, and *nifK* showed relatively high abundances in the middle and peat layers in August. This pattern may be associated with the relatively high temperature and favorable moisture conditions in August, which may have promoted microbial metabolic activity. Meanwhile, increased plant N demand during the rapid growth stage of maize may have altered soil N availability, thereby affecting the abundances of genes involved in N fixation. In addition, the high organic matter content and moisture level, together with the relatively reducing conditions in the peat layer, may have provided suitable habitats for some anaerobic or facultatively anaerobic N-fixing microorganisms, thereby contributing to the enrichment of N-fixation genes [36].

Genes involved in nitrification also exhibited pronounced temporal differentiation, suggesting potential niche partitioning between microorganisms involved in ammonia oxidation and nitrite oxidation in cool-climate maize fields. During maize maturation in September and October, SOM mineralization may have increased ammonium supply and turnover, potentially favoring the enrichment of *amoA*, *amoB*, and *amoC* [37]. The relatively high temperature and favorable moisture conditions in August may have enhanced hydroxylamine oxidation, thereby contributing to the increased abundance of *hao* [38]. Under the relatively low temperatures in May, cold-adapted nitrite-oxidizing bacteria may have gained a competitive advantage, resulting in a relatively high abundance of *nxrA* [39].

The assimilatory nitrate reduction genes *narC* and *NR* showed relatively high abundances in September and October, indicating that soil microorganisms in cool-climate maize fields may preferentially assimilate nitrate into organic N during maize maturation, thereby promoting organic N storage [40]. In the peat layer in August, the abundances of genes involved in DNRA varied considerably. The high C/N ratio and strongly reducing environment of the peat layer may have inhibited the initial step of denitrification, resulting in relatively low abundances of nar-family genes [41,42]. In contrast, the environment characterized by high temperature and moisture, strong anaerobic conditions, and a high C/N ratio may favor the reduction of nitrite to ammonium. In addition, the high substrate affinity of the nitrite reductase encoded by *nrf* genes may allow this process to proceed efficiently even under low-nitrite conditions. Together, these factors may ultimately promote the enrichment of *nrf*-family genes [43].

The pronounced temporal differentiation of genes involved in anammox suggests that anammox bacterial communities in cool-climate maize fields may exhibit niche differentiation associated with temperature adaptation [44]. Among these genes, *hzsA* showed a relatively high abundance in May, suggesting that some anammox bacteria may be cold tolerant and maintain the functional potential for hydrazine synthesis under the low-temperature conditions of early spring, thereby potentially increasing the risk of N loss [45].

### 4.4. Regulatory Mechanisms of Soil Environmental Factors on Nitrogen-Cycling Microbial Communities and Functional Genes

Mantel tests further revealed potential associations between soil environmental factors and microbial communities. Bacterial community composition was significantly correlated with both the MBC/MBN ratio and NFR. The significant association between bacterial community composition and NFR suggests that variation in bacterial community structure may be closely linked to soil N fixation in cool-climate maize fields.

Previous research has shown that fungi play important roles in organic matter decomposition and nutrient release and that fungal communities are generally sensitive to changes in soil resources and environmental disturbances [46]. Changes in fungal community structure and diversity may, in turn, influence soil N-cycling processes. In the present study, fungal community diversity was significantly correlated with MBC, MBN, SW, and NA, indicating that soil moisture conditions and soil resource availability are also important environmental factors associated with variation in fungal communities in cool-climate maize fields.

In cool-climate maize fields, N-cycling functional genes exhibited markedly different responses to soil environmental factors. The abundances of genes involved in NF were positively correlated with NO_3_^−^-N but showed no significant association with NH_4_^+^-N. This finding differs from the conventional view that high nitrate availability suppresses N fixation [47]. Cool-climate agricultural soils have relatively high SOM contents. SOM mineralization can provide C substrates for N-fixing microorganisms and may also promote NO_3_^−^-N accumulation. Therefore, this positive correlation more likely represents an indirect association arising from coupled soil C and N dynamics rather than a direct stimulatory effect of NO_3_^−^-N on N fixation [48].

Both MBC and SW were positively correlated with the abundance of *narZ* but negatively correlated with that of *napA*. Higher MBC and SW in cool-climate maize fields may create high-C, oxygen-limited, or anaerobic microsites that favor the enrichment of *narZ*, causing DNRA to rely more strongly on the Nar enzyme system [40,41]. The periplasmic nitrate reductase encoded by *napA* is generally better adapted to low-C or microaerobic environments [43]. Under high-C and anaerobic conditions, *napA* may be suppressed by environmental factors and interspecific competition, accounting for its negative correlations with MBC and SW. These results suggest that DNRA may be dominated by the Nar rather than the Nap enzyme system under high-C and anaerobic conditions.

In addition, *nrfC* abundance was positively correlated with NFR, suggesting a potential synergistic N-retention effect in cool-climate maize fields [11,49]. N fixation introduces external N, whereas DNRA reduces N loss; together, these two ammonium-producing pathways may help maintain the stability of the soil ammonium pool in cool-climate maize fields.

The anammox genes *hzsA* and *hzo* were positively correlated with MBC but negatively correlated with NO_3_^−^-N, suggesting that these genes may be more readily enriched in microhabitats characterized by active C metabolism and relatively low nitrate availability in cool-climate maize fields. In addition, active heterotrophic microbial metabolism under high-C conditions may accelerate oxygen consumption, thereby creating suitable oxygen-limited or anoxic conditions for anammox [50].

PLS-PM indicated that soil physicochemical properties had a positive effect on bacterial community composition, whereas bacterial community composition had a negative effect on N-cycling functional genes. More favorable physicochemical conditions may shift bacterial communities toward copiotrophic taxa with r-selected life-history strategies, whereas some N-cycling functional genes may be more prevalent among oligotrophic taxa with K-selected strategies [51,52]. Consequently, an increase in copiotrophic bacteria may dilute the relative abundances of oligotrophic taxa and their associated functional genes. These findings suggest that the spatiotemporal differentiation of N-cycling functional genes may be shaped not only directly by environmental factors but also indirectly by trade-offs among bacterial life-history strategies [53,54], rather than exhibiting a simple positive relationship with overall bacterial community complexity.

## 5. Conclusions

This study revealed the spatiotemporal differentiation of soil environmental factors, microbial communities, and N-cycling functional genes in cool-climate maize fields, as well as the potential mechanisms underlying their associations. Soil environmental factors exhibited significant seasonal variation and vertical differentiation, with SOM and TN increasing with soil depth. The dominant form of soil inorganic N shifted from NH_4_^+^-N during the early stage of maize growth to NO_3_^−^-N during the middle and late growth stages. NFR, NitR, and DNR all peaked in August and exhibited a spatial pattern characterized by N fixation in the lower layer and denitrification in the upper and middle layers.

Bacterial and fungal communities responded differently to temporal and vertical gradients. Bacterial communities remained relatively stable, whereas fungal communities exhibited greater fluctuations in diversity and species composition. The genes *nifK*, *hao*, *nirS*/*nirK*, *NR*, *nrfC*, and *hzsA*/*hzsC* were identified as key functional genes involved in different N-cycling processes. Mantel tests and PLS-PM further indicated that soil physicochemical properties may be indirectly associated with N transformation rates and key enzyme activities by affecting bacterial community composition and, subsequently, N-cycling functional genes.

## Figures and Tables

**Table 1:** Soil physicochemical indexes and nitrogen conversion rate analysis methods.

Analytical Indicators	Analytical Methods
pH	Portable soil pH meter
T	Portable soil thermometer
SW	Gravimetric oven-drying method
SOM	Loss-on-ignition method
TN	Semi-micro Kjeldahl method [21]
NO_3_^−^-N	Calcium chloride extraction-spectrophotometry [21]
NO_2_^−^-N	Calcium chloride extraction-spectrophotometry [21]
NH_4_^+^-N	Nessler’s reagent colorimetry
MBC	Fumigation-extraction and volumetric analysis [22]
MBN	Fumigation-extraction and volumetric analysis [22]
NFR	Acetylene reduction assay [23]
NitR	Soil incubation method
DNR	Acetylene inhibition method
NA	Sandwich Enzyme-Linked Immunosorbent Assay
NIO	Sandwich Enzyme-Linked Immunosorbent Assay
DE	Sandwich Enzyme-Linked Immunosorbent Assay

## Data Availability

The datasets generated during and analysed during the current study are available from the corresponding author on reasonable request.

## Abbreviations

The following abbreviations are used in this manuscript:

SOM	Soil organic matter
TN	Total nitrogen
NH_4_^+^-N	Ammonium nitrogen
NO_3_^−^-N	Nitrate nitrogen
NO_2_^−^-N	Nitrite nitrogen
ANR	Assimilatory nitrate reduction
DNRA	Dissimilatory nitrate reduction to ammonium
T	Soil temperature
SW	Soil water content
MBC	Microbial biomass carbon
MBN	Microbial biomass nitrogen
NFR	Nitrogen fixation rate
NitR	Nitrification rate
DNR	Denitrification rate
NA	Nitrogenase activity
NIO	Nitrite oxidase activity
DE	Denitrifying enzyme activity
NCycDB	Nitrogen Cycling Database
PCoA	Principal coordinates analysis
VPA	Variation partitioning analysis
PCA	Principal component analysis
PLS-PM	Partial least squares path modeling
NF	Nitrogen fixation
GoF	Goodness of fit
anammox	Anaerobic ammonium oxidation
